# Integrating morphological characters, molecular markers, and distribution patterns to assess the identity of *Blepharis* species from Jordan

**DOI:** 10.1186/s40529-018-0234-x

**Published:** 2018-07-25

**Authors:** Riyadh Muhaidat, Mohammad H. Brake, Mazhar Al Zoubi, Robert I. Colautti, Amjad Al-Nasser, Muheeb Awawdeh, Khalid Al-Batayneh, Wesam Al Khateeb, Athena D. McKown, Jamil Lahham, Ahmad El-Oqlah

**Affiliations:** 1 Department of Biological Sciences, Faculty of Science, Yarmouk University, P. O. Box 21163, Irbid, Jordan; 2 Science Department, Faculty of Science, Jerash University, Jerash, Jordan; 3 Department of Basic Sciences, Faculty of Medicine, Yarmouk University, P. O. Box 21163, Irbid, Jordan; 4 Department of Biology, Queen’s University, 116 Barrie Street, Kingston, ON K7L 3N6 Canada; 5 Department of Statistics, Faculty of Science, Yarmouk University, P. O. Box 21163, Irbid, Jordan; 6 Department of Environmental and Earth Sciences, Faculty of Science, Yarmouk University, P. O. Box 21163, Irbid, Jordan; 7 Department of Forest and Conservation Sciences, Faculty of Forestry, University of British Columbia, Forest Sciences Centre, 2424 Main Mall, Vancouver, BC V6T 1Z4 Canada

**Keywords:** *Blepharis attenuata*, *Blepharis ciliaris*, Inflorescence, Spike, Bract, Dimorphic stamens, Linear discriminant analysis, Inter-Simple Sequence Repeat

## Abstract

**Background:**

*Blepharis* constitutes an important part of the vegetation of the Jordanian arid and semi-arid regions, yet whether one or more species of this genus occurs in the Jordanian area is uncertain. We addressed this question by assessing morphological characters and testing Inter-Simple Sequence Repeat (ISSR) markers from three populations of *Blepharis*: two northern (lower slopes of Kufranjah valley and the Dead Sea region) and one southern (Wadi al Yutm).

**Results:**

Shoots from randomly chosen *Blepharis* plants were harvested from each of the three populations for morphological and molecular analyses. In the northern populations, spikes were lax and bract width was significantly shorter than length of the longest lateral spine compared to the southern population. A multivariate linear discriminant analysis distinguished the northern populations from the southern one by internode length, bract width, longest lateral spine length, and bract width to spine length ratio. The ISSR analysis revealed that 44 markers across eight primers were polymorphic with major allele frequency of 83.6% and an average of 5.5 polymorphic markers per primer. The genetic resemblance among individuals ranged from 0.27 to 0.96. The three *Blepharis* populations were accordingly clustered into two distinct groups, similar to the analysis of morphological differences and corresponding with the “northern” and “southern” population designations.

**Conclusions:**

Our results strongly indicate the occurrence of two discrete *Blepharis* species in Jordan and reject the hypothesis that the genus is represented by only one species. We propose that the *Blepharis* species in Jordan are *B*. *attenutata* Napper (represented by the northern populations) and *B*. *ciliaris* (L.) B. L. Burtt (represented by the southern population). These findings are important for informing and revising floristic work within the region and an updated key has been included in our findings.

## Background

*Blepharis* Juss. is a genus in the family Acanthaceae s.l. (subfamily Acanthoideae, tribe Acantheae) comprising 129 species that are broadly distributed in hot, arid and semi-arid regions of the old world tropics and subtropics, with the center of diversity in eastern and southern Africa (Vollesen 2000, 2002; Sage 2004; Fisher et al. 2015). In the Middle East, including Jordan, *Blepharis* constitutes an important component of the desert vegetation. The genus is highly specialized to inhabit extreme desert environments that receive little rain in winter and experience high temperatures and light intensities during summer (Gutterman 2002). In the Middle East region, two species of *Blepharis* were originally recorded and described in regional floras, and both were regarded as distinct. These included *B*. *attenuata* Napper and *B*. *ciliaris* (L.) B. L. Burtt (Boulos and Lahham 1977; Feinbrun-Dothan 1978; Gutterman 2002). Yet, debate over the number and identify of *Blepharis* species occurring in this region has arisen following the taxonomic revision of the genus by Vollesen (2000). Based largely on assessments of herbarium materials, Vollesen (2000) argued that *B*. *attenuata* is the sole member that migrated north and colonized the desert areas of Egypt, Israel and West bank, and Jordan. Vollesen (2000) also considered *B*. *attenuata* as the only species in the Acantheae that grows below the sea-level.

In a survey of leaf anatomy and photosynthetic biochemistry in C_4_ species, Muhaidat et al. (2007) identified *Blepharis* samples from Jordan as *B. ciliaris*. Akhani et al. (2008) brought attention to the possible misidentification of *Blepharis* from Jordan in the literature and floras of the Middle East. A subsequent study by Muhaidat et al. (2012) identified plants collected from the lower parts of the Jordan valley as *B. attenuata* through morphological characters relating to the general growth habit and inflorescence (Feinbrun-Dothan 1978; Vollesen 2000). None of these studies, however, were able to clearly identify in a comparative context whether one or more *Blepharis* species exist within the Jordanian flora.

**Fig. 1:**
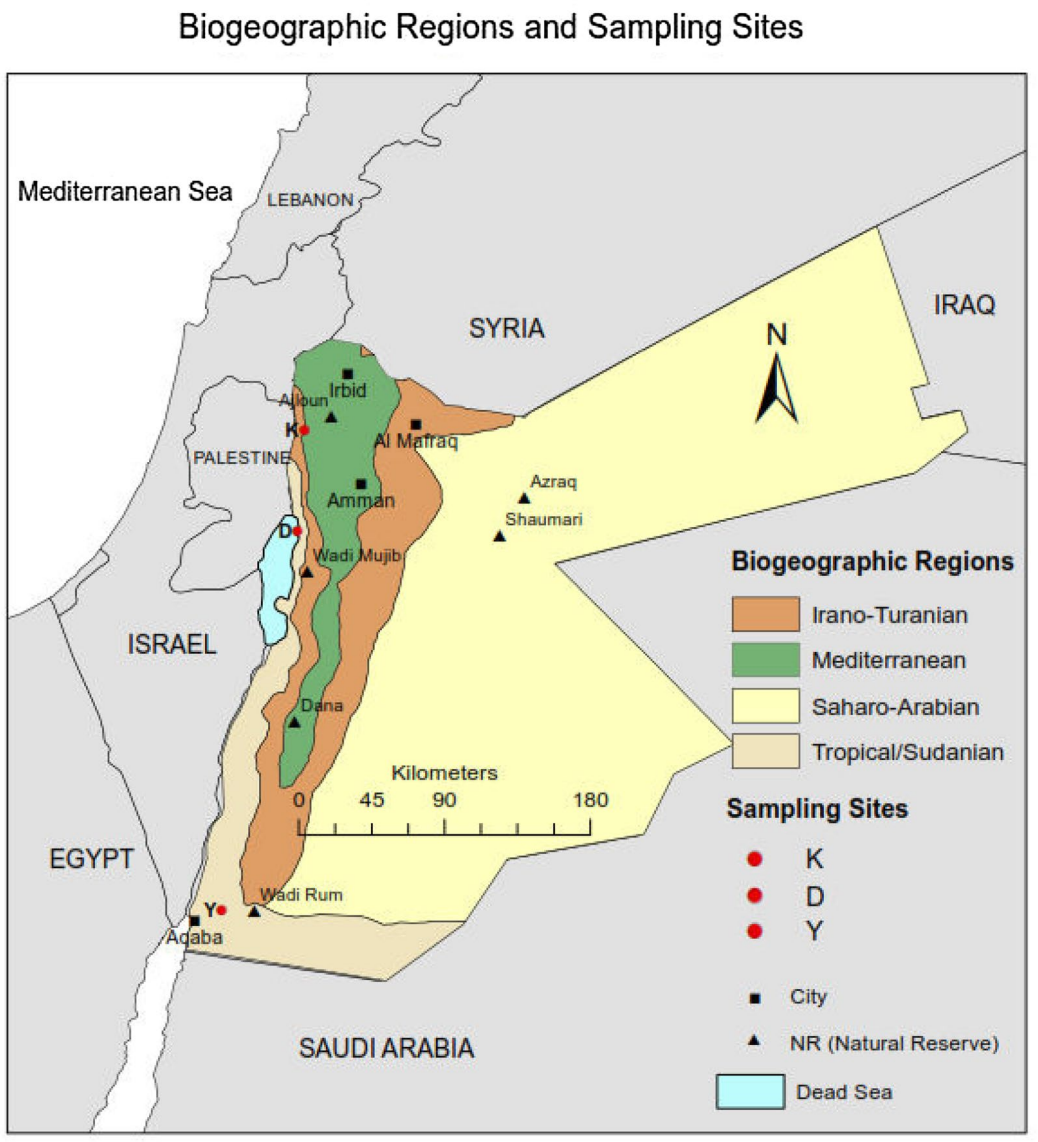
Map of Jordan biogeography and locations of sampling sites for *Blepharis*. Sampling sites are indicated by red circles. K, Kufranjah valley; D, Dead Sea region; Y, Wadi al Yutm

In Jordan, the distributional range of *Blepharis* is ecologically broad. The genus occurs along desert, rocky slopes and wadi beds (dry riverbeds) of west Irano-Turannian, east Saharo-Arabian and Sudanian (or tropical penetration) biogeographical regions (Fig. 1). These biogeographical zones of Jordan were circumscribed based on variations in altitude, temperature regimes, amount and patterns of rainfall, types and textures of soils, and the very diverse flora of the region (Long 1957; Feinbrun-Dothan 1978; Elesawi 1985; Palmer 2013). The ecological and climatic diversity offers a great opportunity to explore and assess *Blepharis* species identities in relation to geography as a test region for the Middle East. If a single *Blepharis* species occurs in Jordan, then it would exist across multiple types of environments, as is the case for some plant species in the region. By comparison, multiple *Blepharis* species in Jordan may indicate biogeographical preference or habitat specialization.

There is a striking diversity of morphological characters in *Blepharis*, with life forms ranging from annual and perennial herbs to subshrubs and shrubs (Vollesen 2000). Characters related to habits, inflorescences, and floral characteristics were regarded the most useful for discerning *Blepharis* species (Vollesen 2000). The genus is distinctive in having inflorescences ranging from spikes (or pseudo-spikate cymose) to a reduced cyme or a solitary terminal flower and leaves are arranged in a pseudo-whorled phyllotaxy. Bracts are leathery (coriaceous) with spiny margins or thin and papery (glumaceous) with either entire or bristly margins. Bracteoles, when present, are linear to lanceolate with bony midribs, glumaceous and generally have entire margins. Stamens are notably dimorphic and occur as an anterior and posterior pairs. The anterior pair bears laterally flattened filaments, is hairy toward the base, and appendaged distally, while the posterior pair is slightly narrower with linear filaments that are “knee-like” and unappendaged. Pistils of *Blepharis* flowers possess lobed stigma, styles that are filiform, glabrous (or rarely hairy with tufty basal glandular trichomes), and a bilocular ovary bearing two ovules per locule. Fruits are ellipsoid capsules that are lignified and explosively dehiscent, enclosing flat seeds coated with hygroscopic branched hairs borne on hook-like structures called retinacula (Vollesen 2000; Gutterman 2002).

Feinbrun-Dothan (1978) outlined a number of diagnostic and discriminatory morphological traits among *Blepharis* taxa and used these in a taxonomic key for distinguishing species. The laxness of the inflorescence, length and width of bracts, number of bract veins and lateral spines, length of the longest lateral spine, and the ratio of spine length to bract width were noted as important characters for identifying *B. attenuata* and *B. ciliaris*. Variability in morphological features of bracts and the degree of spikes compactness were particularly important in distinguishing between *B. attenuata* and *B. ciliaris* and were highlighted in the taxonomic key (Feinbrun-Dothan 1978). To the best of our knowledge, further assessments of these diagnostic morphological features have not been pursued.

The purpose of this study was to clearly identify the species of *Blepharis* in Jordan and to test whether the hypothesis that only one species of the genus occurs in the region is supported. We assessed morphological features, including those previously regarded as distinctive characters (Feinbrun-Dothan 1978; Vollesen 2000), and used linear discriminant analysis (LDA) to address differentiation of three distinct populations of *Blepharis* in this study. The morphological analyses were complemented by Inter-Simple Sequence Repeat (ISSR) analysis and non-metric multidimensional scaling (NMDS) to compare possible morphological and genetic variation. Compared to Random Amplified Polymorphic DNA (RAPD), the ISSR methodology has been used successfully for its specificity, reproducibility, high stringency, and compatibility in gene mapping, genome fingerprinting studies, cultivar identification, and assessing genetic diversity and relatedness among closely related species and landraces (Bornet and Branchard 2001; Galván et al. 2003; Serra et al. 2007; Al Khateeb et al. 2013; Khierallah et al. 2014; Brake et al. 2014; Ng and Tan 2015; Salazar-Laureles et al. 2015; Yuan et al. 2015; Costa et al. 2016; Chaubey et al. 2017; Sheng et al. 2017). Using the combined morphological assessments and ISSR molecular markers, our study is the first to propose which species of *Blepharis* occur in Jordan. An updated key for both *Blepharis* species is included in this study and notes on their distribution and ecology were taken into account. We consider that it is important to correctly assess the identity of *Blepharis* species in the Middle East so that local floristic surveys can be revised and future errors may be avoided.

## Materials and methods

### Sampling

Three populations of *Blepharis* in Jordan were used for morphological characterizations and molecular genotyping. These sampling sites were chosen where the genus was recorded and observed to be abundant (Fig. 1). The three sites included: lower slopes of Kufranjah valley in the uppermost portion of the west Irano-Turannian region (32°14.872′N; 35°37.007′E, altitude—169 m below sea level), the Dead Sea area in the tropical/Sudanian region (31°41.364′N; 35°34.736′E, altitude—397 m below sea level), and Wadi al Yutm in the southern segment of the Sudanian/tropical region, 25–30 km NE of Aqaba city (29°35.098′N; 35°09.462′E, altitude 623 m above sea level). The first two sampling sites (“K” and “D”, Fig. 1) were located in the Jordan valley (Al-Ghor, the narrow trough of the lower course of the Jordan river), which forms part of the larger Jordanian rift valley, and were designated as the ‘northern populations’. The third sampling site (“Y”, Fig. 1) was located in Wadi Arabah or Arava valley, the portion of the rift valley beyond the Dead Sea and ending at Aqaba/Eilat farther south, and was designated as the ‘southern population’.

**Fig. 2:**
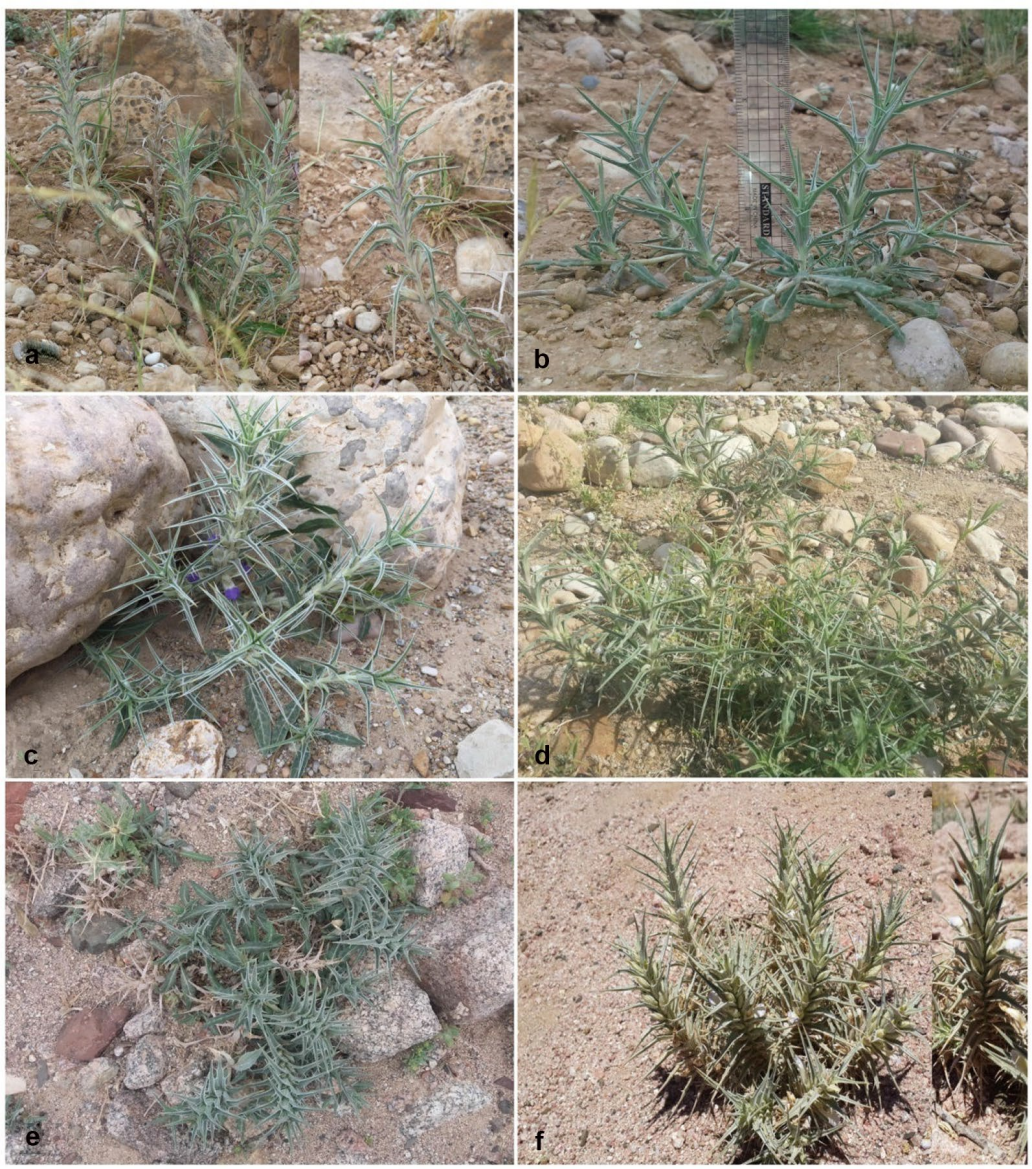
Wild accessions of *Blepharis* within their natural habitat in sites of collection in Jordan showing their spiny growth habits, spike-type inflorescence, and few basal leaves. **a**, **b** Natural habit in Kufranjah valley (northern population). **c**, **d** Natural habit in the Dead Sea region (northern population). **e**, **f** Natural habit in Wadi al Yutm (southern population). Dead spikes from prior years on plants of recent growth are apparent in **a**, **d**, **e**. Note also green spike overtopping old capsule-containing spike in **d**

At each site, *Blepharis* plants were readily recognized from their growth habit, pseudo-whorled leaves, and inflorescence structure. Plants were all perennial herbs and growing on stony hilltops, desert slopes, and in wadi beds on rocky grounds, runnels and fissures (Fig. 2). Mature shoots were collected from 19 randomly selected *Blepharis* plants from each site during April 2013 and used for morphological characterization. Young leaves were harvested from the same plants for molecular genotyping, dried in silica gel, and preserved at − 20 °C until use. Voucher specimens from all sites were deposited in the Herbarium at the Department of Biological Sciences, Yarmouk University (herbarium specimens # 35-2013, # 36-2013, and # 37-2013 for Kufranjah, Dead Sea, and Wadi al Yutm populations, respectively [collectors: Muhaidat, El-Oqlah, Lahham, Al Khateeb]).

### Morphological analysis

**Fig. 3:**
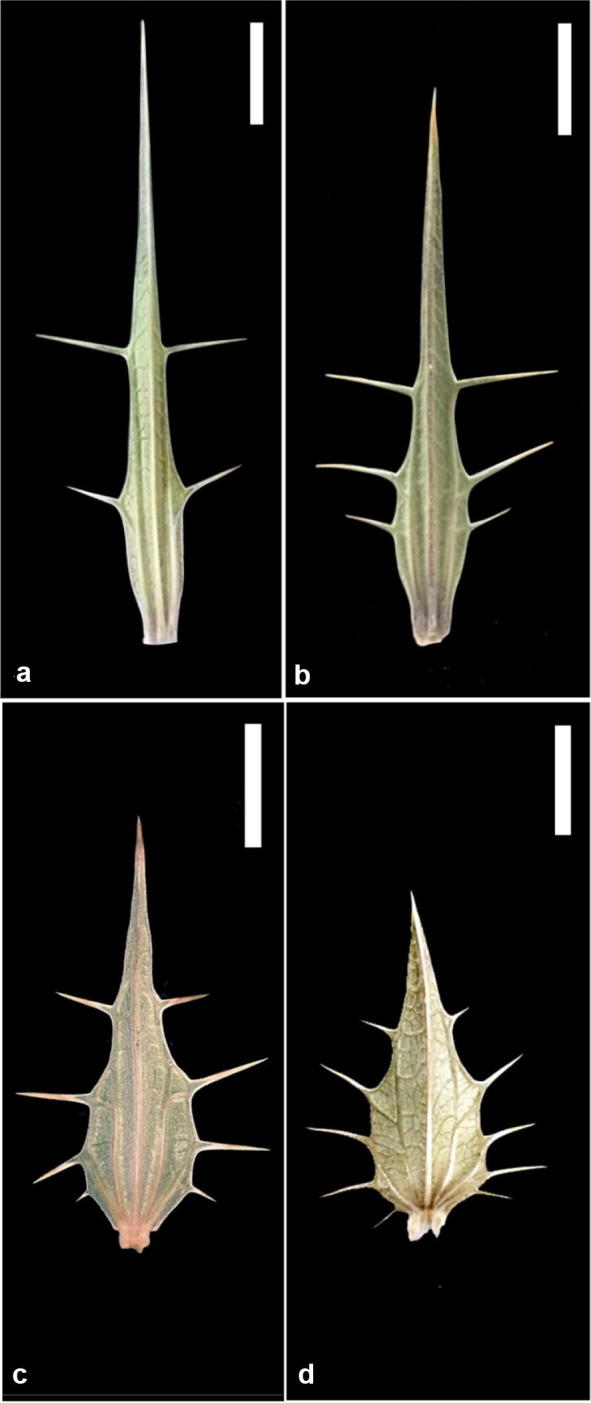
Mature bracts from *Blepharis* plants collected from northern and southern populations in Jordan highlighting differences in numbers of veins and numbers and lengths of the stout lateral spines. **a**, **b** Bracts of a plant from the Dead Sea region (northern population). **c**, **d** Bracts of a plant from Wadi Al Yutm (southern population). Scale bars = 1 cm

**Fig. 4:**
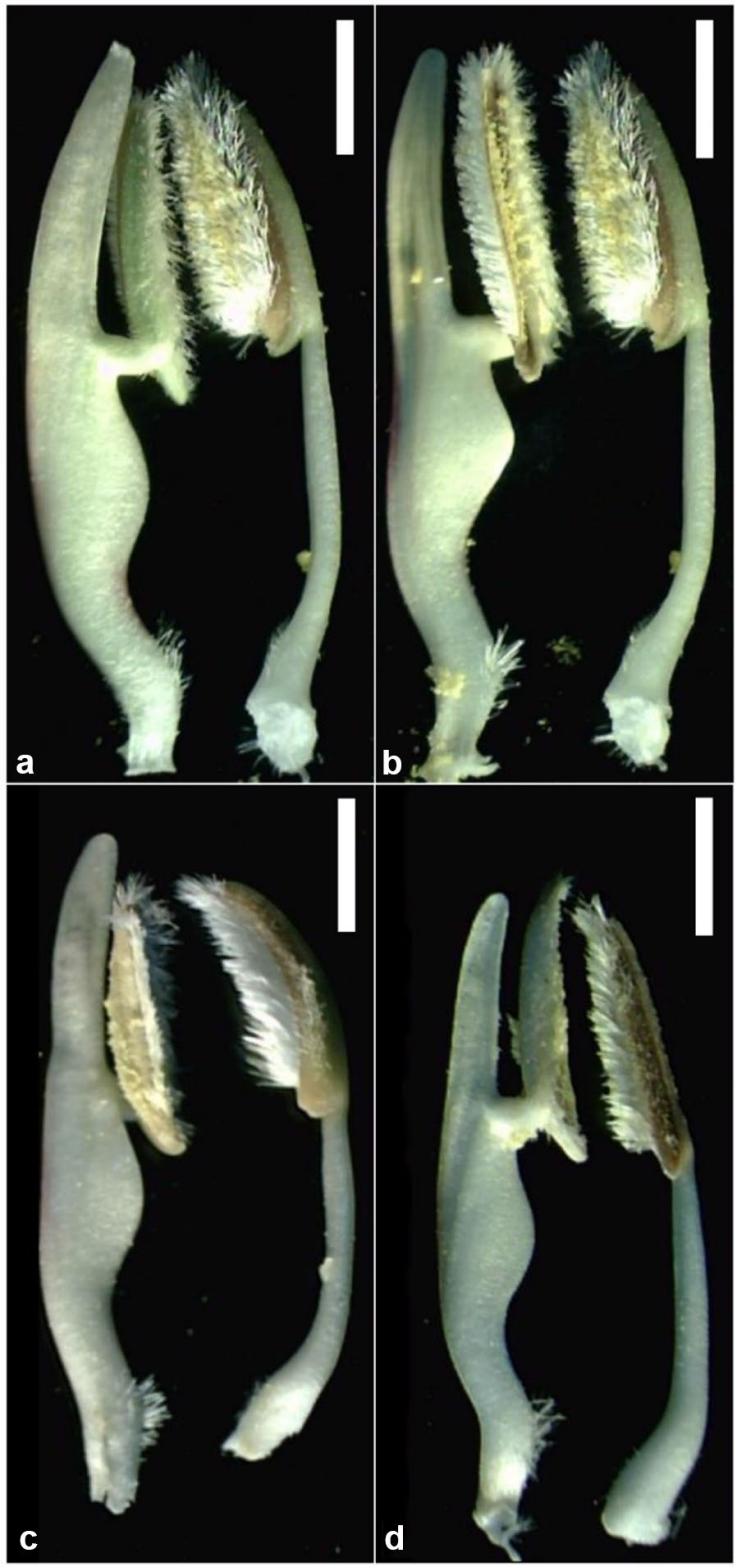
Dimorphic stamens with “knee-like” appendages on the filaments from *Blepharis* plants collected from northern and southern sampling sites in Jordan. **a**, **b** Stamens of a plant from the Dead Sea region (northern population). **c**, **d** Stamens of a plant from the Wadi al Yutm (southern population). Each stamen pair represents an anterior (left) and a posterior (right) stamen. Scale bars = 2 mm

Shoots collected from the three *Blepharis* populations were measured for morphological characters previously regarded as diagnostic and distinguishing features at the species level (Feinbrun-Dothan 1978; Vollesen 2000). Quantified variables included floral bract length and width (Fig. 3), number of veins per bract, number of lateral spines per bract margin, length of the longest lateral spine, ratios of the longest spine length to bract width, distance between successive flowering spikelets (termed henceforth as “internode length”), leaf length and width, number of teeth per leaf margin, filament and anther lengths for each of the dimorphic anterior and posterior stamens, and appendage length for the anterior stamens (Fig. 4). Stamens were measured under a dissecting microscope (Wild Heerbrugg, Switzerland) and photographed using an eyepiece digital camera (Optika digital camera 4083.B9 and OptikaISview v3.6.6 software: Optika Digital Microscopy, Ponteranica, Italy).

Linear discriminant analysis (LDA) as implemented in the MASS package (ver. 7.3–45) in R (ver. 3.3.2) was used to determine morphological differences among the studied populations considering all measured variables simultaneously (Venables and Ripley 2002). Variables were transformed to a mean of zero and unit standard deviation before analysis. Morphological differences among the three *Blepharis* populations were tested used basic linear models (*lm*) in the standard R package with each LDA axis as a response variable and region of origin as the predictor. We also used the *lm* function to test whether individual traits differed among geographical regions and a multiple pairwise comparisons test (Scheffe test) was implemented for the significant results obtained in the LDA. Detailed methods with raw data and original R code are available from the Dryad Digital Repository (10.5061/dryad.66jn7g7).

### Molecular methods

#### DNA extraction and ISSR fingerprinting

Total genomic DNA was successfully extracted from 56 individuals (19, 18, and 19 plants from Kufranjah (K1–K19), Dead Sea (D1–D18), and Wadi al Yutm (Y1–Y19) populations, respectively) using a Gene JET Plant Genomic DNA Purification Kit (Thermo Fisher-SCIENTIFIC, MA, USA). Silica-dried leaf samples were ground to a fine powder in liquid nitrogen using a pre-chilled mortar and pestle. DNA was extracted following the manufacturer’s instructions, and DNA quantity and quality was evaluated using both a Nanodrop Spectrophotometer (Thermo Fisher-SCIENTIFIC, MA, USA) and on a 1% agarose gel with electrophoresis.

ISSR fingerprinting analysis was performed following Bornet and Branchard (2001) using eight primers (Midland Certified Reagent Company, Inc., USA) (Table 1) according to the primer set published by University of British Columbia, Canada (Al Khateeb et al. 2013; Brake et al. 2014). Prior to this study, these primers had not been used in *Blepharis*. A few samples were initially tested using 45 primers. Those generating high levels of DNA polymorphism were chosen and used for all 56 samples that represent the three *Blepharis* populations. Amplification was carried out in 25 μl reaction mixture contained 30 ng of genomic DNA, 0.3 μM of the primer, 1X Taq DNA polymerase reaction buffer, 1.5 unit of Taq DNA polymerase and 0.2 mM of each dNTP. Amplifications were performed in Bioer-XP thermal cycler (BIOER, Hangzhou, China) programmed for an initial denaturation step of 5 min at 94 °C, followed by 45 cycles composed of 30 s at 94 °C, 45 s annealing at 52 °C, and 90 s at 72 °C, and a final extension of 72 °C for 5 min. Amplified products of ISSR were separated on 1.5% agarose gels in 1X TBE buffer. Fragment size was estimated manually relative to Quick-Load^®^ 100 bp DNA Ladder (New England BioLabs Inc., Ipswich, MA, USA) and detected by staining with ethidium bromide (10 mg/ml) according to Sambrook et al. (1989). The PCR products were visualized by UV-transilluminator and photographed using a gel-documentation system (BioDocAnalyze: Biometra, Jena, Germany).

**Table 1:** Linear discriminant (LD) axis loadings of 14 morphological characteristics measured in *Blepharis* plants from three populations in Jordan

Trait	LD axis 1	LD axis 2	*F*	*P* value
Leaf				
Length	− 0.068	0.211	1.9	0.161
Width	− 0.140	− 0.687	0.4	0.644
# Teeth per margin	− 0.120	− 0.272	0.2	0.806
Spike				
Internode length	2.007	1.249	*169.3*	< 0.001
Bract length	0.628	− 0.450	*20.6*	< 0.001
Bract width	− 1.717	1.076	*134.7*	< 0.001
# of veins per bract	− 0.352	0.257	*14.9*	< 0.001
# of lateral spines per bract side	− 0.017	− 1.296	*57.3*	< 0.001
Length of the longest lateral spine	− 0.081	− 1.335	*43.9*	< 0.001
Anterior stamen				
Filament length	− 0.284	− 0.873	1.6	0.209
Anther length	− 0.040	− 0.190	0.1	0.944
Appendage length	0.149	0.466	1.3	0.274
Posterior stamen				
Filament length	0.282	0.429	0.02	0.983
Anther length	− 0.058	0.142	0.6	0.528

Larger *F* values represent stronger differentiation among *Blepharis* groups and significant *P* values are in italics

#### Genetic data analysis

For each primer used to assess *Blepharis* samples, the gel was analyzed by scoring the presence or absence of ISSR bands. The presence of an amplified fragment was scored as “1”, while its absence was scored as “0” (Khierallah et al. 2014; Brake et al. 2014; Ng and Tan 2015). Discrimination power was calculated by dividing the polymorphic markers produced from each primer by the total polymorphic markers produced. Data obtained by scoring ISSR profiles were used to calculate a similarity matrix using Jaccard’s coefficients. Similarity values were used for subsequent cluster analyses. Sequential agglomerative hierarchical non-overlapping (SAHN) clustering was employed using unweighted pair group method with arithmetic averages (UPGMA) method and dendrograms were plotted using NTSYSpc 2.02 software (Rohlf 1998). Finally, we used nonmetric multidimensional scaling to visualize genotypes. This was implemented in R using the *dist* function from the *proxy* package (v0.4-17) to calculate pairwise Jaccard’s distance, along with the *cmdscale* function (data and analysis files available at 10.5061/dryad.66jn7g7).

## Results and discussion

### General morphology

In their natural habitats in Jordan, all *Blepharis* individuals were strikingly spiny, perennial herbs to subshrubs, with erect, semi-erect to decumbent branches and a few basal leaves at maturity (Fig. 2). The overall habit of the plants easily identified them as belonging to *Blepharis*. Other features from *Blepharis* individuals collected at the three sites in this study identified populations with particular morphological characters corresponding to two species outlined by Feinbrun-Dothan (1978). Although superficially similar (based on general habit), our results strongly suggest the presence of two *Blepharis* species in Jordan: *B. attenuata* occurring in the northern region and *B. ciliaris* occurring in the southern region.

### Diagnostic morphological characters

#### Leaves

Leaves of all *Blepharis* populations investigated in this study were found to be morphologically alike, as previously noted by Vollesen (2000, 2002). Leaves and leaf arrangement were taxonomically useful in recognizing the genus but provided no further value in distinguishing among *Blepharis* species. In all populations, leaves were flat, lanceolate, and leathery, with mostly dentate margins (2–7 teeth per side, rarely more or entire), subsessile to shortly petiolated, with a green color adaxially and whitish–green color abaxially (Fig. 2). The leaf arrangement on all plants in the field was pseudo-whorled, as decussate leaves had shortened internodes to appear as a whorl of four (Feinbrun-Dothan 1978).

#### Inflorescence

All *Blepharis* plants identified in this study had decussate flowers arranged in spikes. Vollesen (2000) argued that the inflorescences of these species are of cymose origin and so described it as pseudo-spicate cyme, but for reasons of ease and brevity, referred this type to as a ‘spike’. In our study, spikes of *Blepharis* individuals from the northern populations were lax (i.e., with longer internodes), whereas those in the southern population were compact, concurring with observations reported earlier by Feinbrun-Dothan (1978) (Fig. 2). As a result, the general spike morphology is supported as a key diagnostic and useful discriminatory character between the explored populations of *Blepharis*. This character was also noted by Feinbrun-Dothan (1978) to distinguish two distinct species: *B. attenuata* with lax spikes and *B. ciliaris* with compact spikes.

In all three populations, newly developed green spikes with different phases of flower and seed development and dead spikes from previous years containing mature capsules were observed on *Blepharis* plants (Fig. 2a, d, e). It has previously been reported that each inflorescence survives for up to one year and later dries out (Gutterman 2002). In our field observations, we noted green fertile spikes re-sprouting distal to older ones or developing from bracts of old inflorescences on *Blepharis* plants from the northern populations but not on those from the southern population (Fig. 2d; see also Muhaidat et al. 2012). This is an additional feature that may be used in distinguishing northern and southern populations.

#### Floral bracts

Flowers on plants from the three *Blepharis* populations were violet, purple, mauve, or rarely white. Each flower was subtended by green, rubbery or coriaceous bract (Figs. 2, 3). Bracts were strikingly recurved, canaliculate (channelled with a longitudinal groove), strongly veined and spiny (Fig. 3). Bracts were observed to be longer, narrower and 3–(5) veined with 2–3 stout, terete, and straight spines on each side in the northern populations (Fig. 3). By contrast, bracts on plants from the southern population were shorter, wider and consistently 5-veined with 3–5 (− 6) spines on each side. The longest spine on bracts from plants in the southern population was notably shorter than any on plants from the two northern populations. These morphological observations are consistent with the variation recorded by Feinbrun-Dothan (1978) outlining two species of *Blepharis*, but inconsistent with Vollesen (2000), who reported that number of spines in *B*. *attenuata* ranged from 2 to 4 (− 5) and 2–3 spines in *B*. *ciliaris*. Reasons for this discrepancy are unclear but might be due to species misidentification, variations among populations from different geographical regions, and/or number of samples investigated.

#### Stamens

Two strikingly dissimilar (dimorphic) anterior and posterior pairs of stamens were noted on plants from all three *Blepharis* populations investigated in this study, and previously reported as diagnostic for the genus (Vollesen 2000; McDade et al. 2005). The anterior pair was laterally flattened, hairy toward the base, and having a flattened appendage distally. The posterior stamens were slightly narrower, curved with knee-like and unappendaged (Fig. 4). Vollesen (1999, 2000) considered the flattened appendage character an advanced character and not present in closely related *Cynarospermum* (Nees) Vollesen (formerly a member of *Blepharis*). In this study, the shape and length of the appendage did not discriminate among the sampled populations, in contrast to other characteristics noted above, and we suggest that the stamen appendage shape and length are not taxonomically useful at the species level.

#### Capsules and seeds

**Fig. 5:**
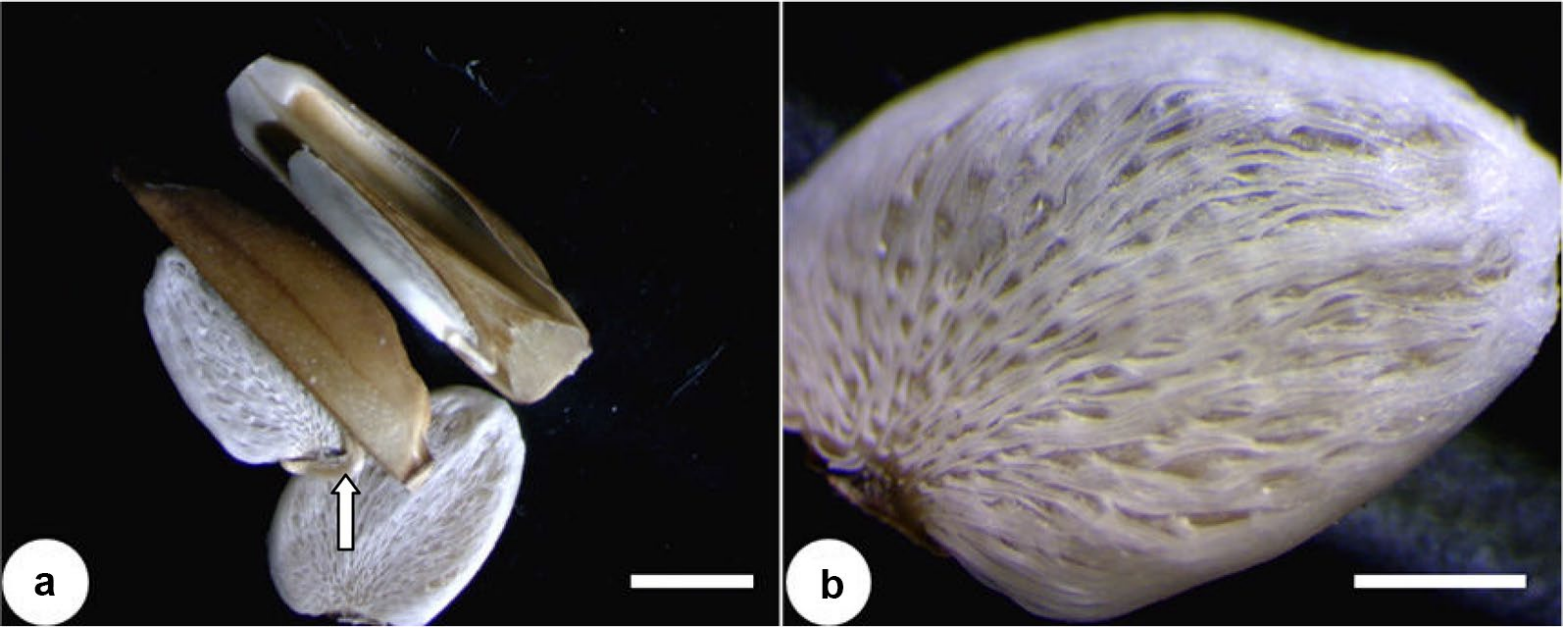
Dry, mature bilocular capsules from a *Blepharis* plant with a pair of seeds on retinacula (arrowhead). **a** Capsule with seeds. **b** Close-up of a flat and ovate seed coated with hygroscopic hairs. Scale bars = 2 mm

Across all the three *Blepharis* populations in our study, the fruits were brown explosively-dehiscent ellipsoid and lignified capsules (Fig. 4). This feature is common to many *Blepharis* species (Feinbrun-Dothan 1978; Vollesen 2000; Gutterman 2002). Each capsule, was 2-loculated, wide-cylindrical, and contain a pair of seeds borne on retinacula that hold the seeds and aids in their dispersal (Fig. 5). Seeds were flat, ovate, acute at one end, rounded at the other end, and coated with white hygroscopic multicellular and branched hairs (Fig. 5). This is consistent throughout the *Blepharis* genus (Vollesen 2000; Gutterman 2002) but did not provide additional informative characters to discriminate between our *Blepharis* populations in Jordan.

#### Statistical analysis of morphological characters

**Fig. 6:**
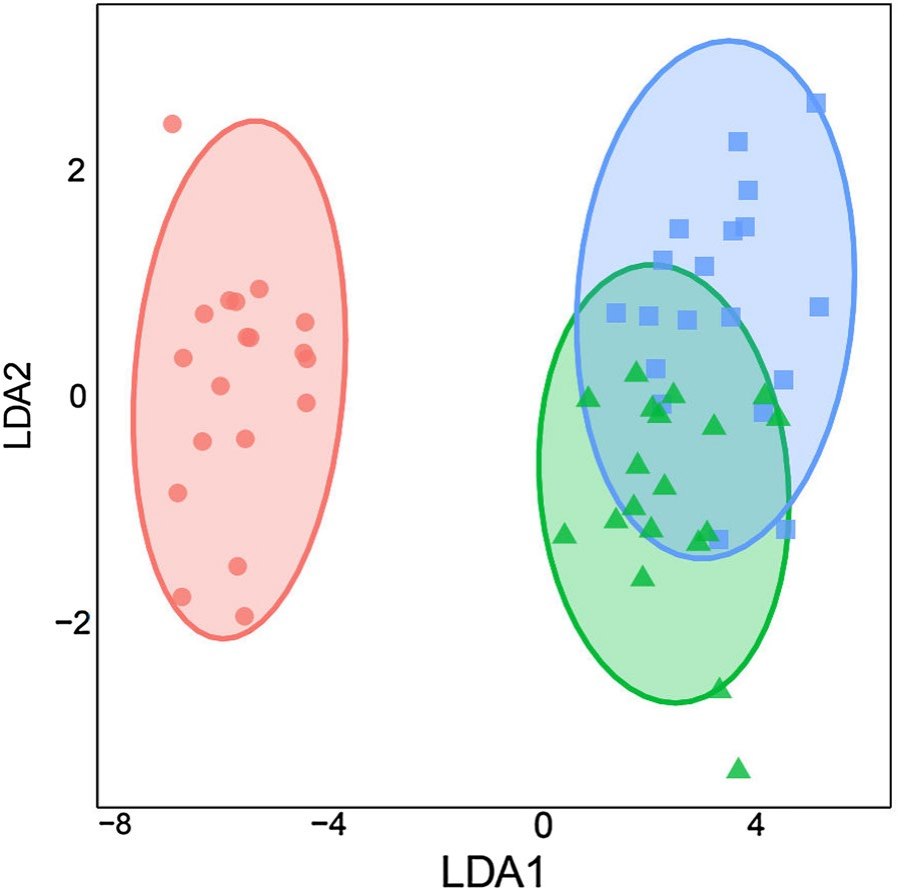
Morphological variation in *Blepharis* populations from Jordan visualized along the first two linear discriminant (LD) axes, and calculated from Euclidean distances of 14 morphological characteristics measured on individuals from Kufranjah valley (northern population, blue squares), Dead Sea region (northern population, green triangles), and Wadi al Yutm (southern population, red circles)

Our LDA using fourteen quantitative morphological variables successfully distinguished *Blepharis* plants collected from the three sites, with a significant difference among group means along axis 1 (*F* = 509.4, *P *< 0.001). In the LDA, northern populations were more similar to each other than either was to the southern population (*F* = 14.0, *P* < 0.001). For these two northern populations, 95% of the ellipses were overlapping (Fig. 6) and could be considered as a single group in the LDA. *Blepharis* plants from the southern population were morphologically distinct and robustly discriminated from those of the northern populations, primarily due to shorter internodes and wider bracts, with no ellipse overlap (Table 1, Fig. 6). The northern and southern populations also differed significantly in bract length, number of veins per bract, and both lateral spines number and length (*P* < 0.001) (Table 1).

**Fig. 7:**
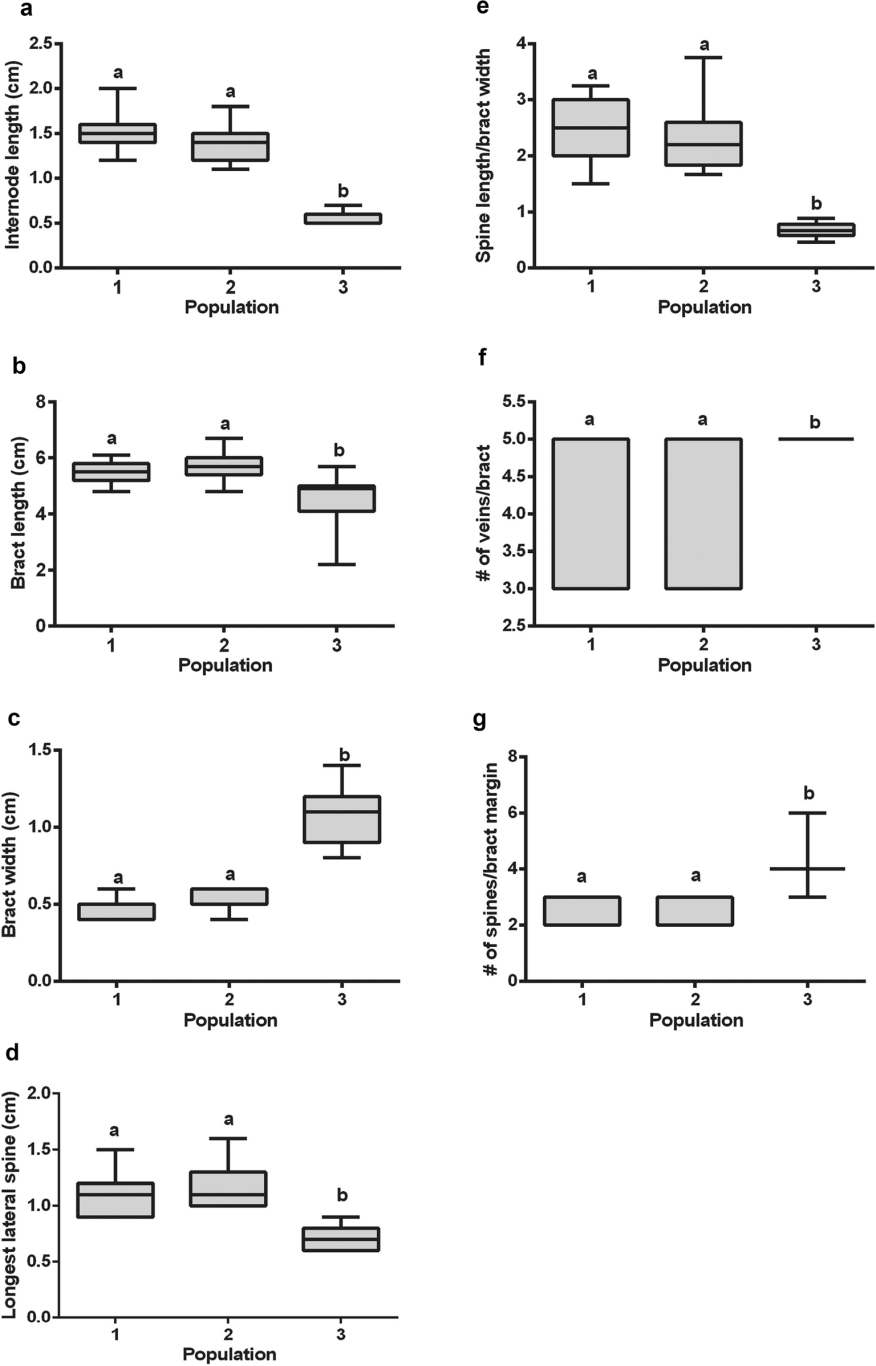
Comparison of data spread of morphological characters amongst *Blepharis* populations sampled at Kufranjah valley (northern population, 1), Dead Sea region (northern population, 2), and Wadi al Yutm (southern population, 3). Graphed variables include **a** internode length (cm), **b** bract length (cm), **c** bract width (cm), **d** longest lateral spine (cm), **e** spine length to bract width ratio, **f** veins per bract (#), and **g** spines per bract margin. Different letters indicate a statistically significant difference at *P* ≤ 0.05 using linear modeling (*lm*). Each box represents 50% of the data and upper and lower box lines represent 75th and 25th percentiles, respectively, of the data set. The line in the middle of the box is the median value of the data, the whiskers indicate the adjusted values

A multiple pairwise comparisons test (Scheffe test) confirmed the significant distinction between the northern and the southern populations obtained in the LDA along the first linear discriminant axis (Table 1, Fig. 7). The primary characteristics of the southern population were shorter internodes (Fig. 7a), shorter but wider bracts (Fig. 7b, c), and shorter lateral spines (Fig. 7d) compared to the plants from the northern populations (*P* < 0.001). This was also reflected in the ratio of spine length to bract width (Fig. 7e), which was markedly lower in the southern population compared to the northern populations (*P* < 0.001). The number of veins and lateral spines/bract margin were considerably greater in the southern *Blepharis* plants than plants from either of the northern populations (Fig. 7f, g) (*P* < 0.001).

Despite the high similarity between the two northern populations based on the LDA, there was not an exact or complete morphological match. Some of this variation may be attributable to the demographic genetics of each population and/or specialization to edaphic conditions, degree of humidity, temperature regimes, patterns and amount of rainfall received at each site (Elesawi 1985; Palmer 2013). Further detailed work is necessary to determine the underlying factors contributing to differences between the northern populations.

#### ISSR analysis

**Fig. 8:**
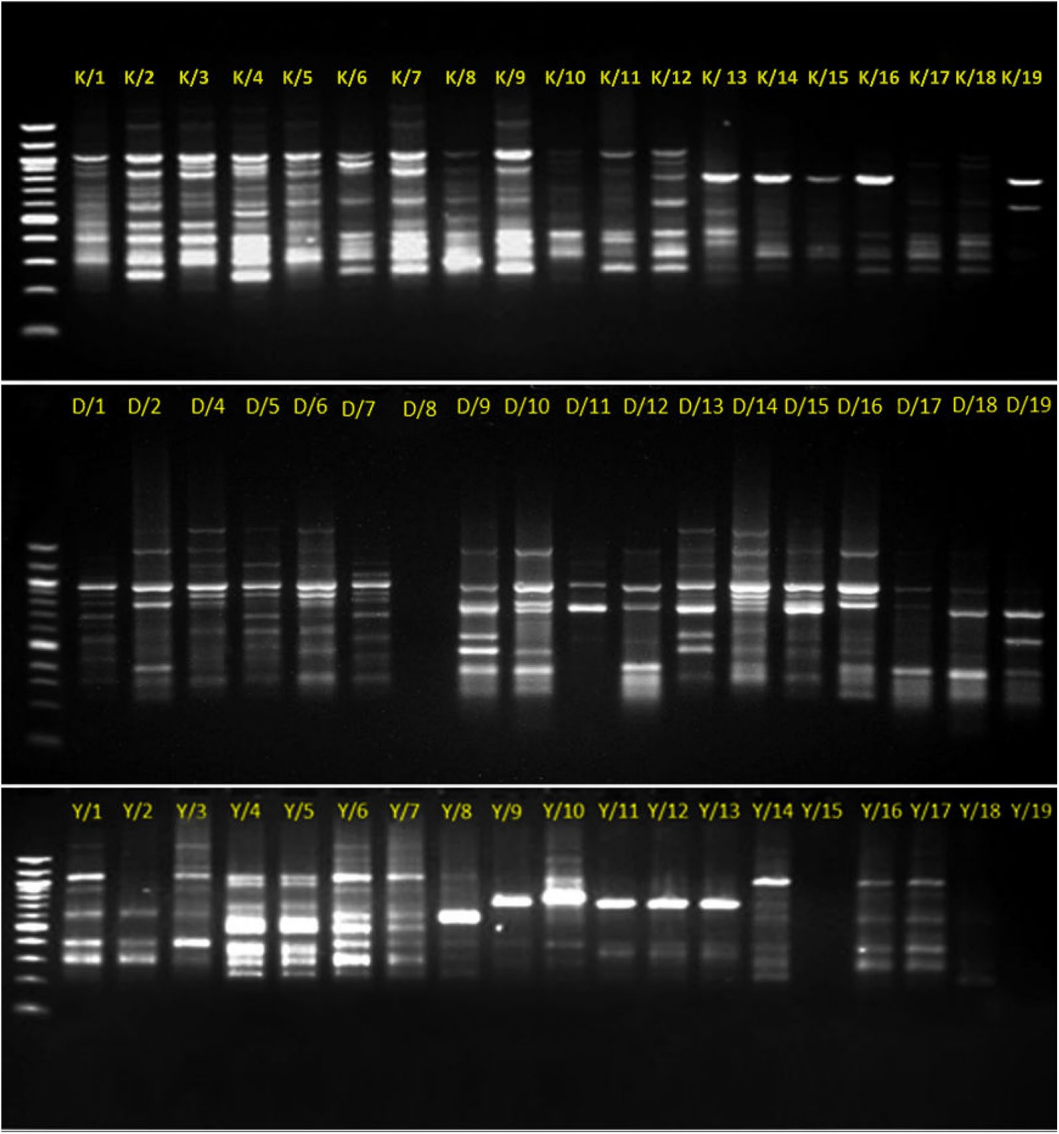
DNA fingerprinting pattern generated by Inter-Simple Sequence Repeats (ISSR) primers of 19 samples (K1–K19) of *Blepharis* from Kufranjah valley (upper image), 18 samples (D1–D19) from the Dead Sea region (middle image), and 19 samples (Y1–Y19) from Wadi al Yutm (lower image). Amplification products were obtained using UBC 848 primer. Accession numbers of each individual are provided at the top of the gel images. D3 accession sample was removed because it showed some discrepancies suggesting the presence of an error

A total of 52 markers ranging from 200 bp to 1500 bp were scored across eight primers, of which 44 markers were polymorphic with major allele frequency < 85% and an average of 5.5 polymorphic markers per primer. Two out of eight primers used showed no polymorphism. The highest discrimination power was observed using the primer UBC 825, while the primers UBC 857, UBC 845, and UBC 809 showed lower discriminating power (Table 2). The amplification profiles of the ISSR analysis were screened for the presence of polymorphisms amongst the sampled *Blepharis* populations (Fig. 8).

**Table 2:** ISSR primers names and sequences, total number of markers, primer efficiency, polymorphic markers, polymorphism, and discrimination power

Primer	Primer sequence 5′–3′	Total markers (#)	Polymorphic markers (#)	Polymorphism (%)	Discrimination power (%)
UBC 857	(AC)_8_GG	6	4	67	9
UBC 864	(ATG)_6_	6	5	83	11
UBC 848	(CA)_8_RG	7	6	86	14
UBC 825	(AC)_7_T	8	8	100	18
UBC 845	(CT)_8_RG	5	4	80	9
UBC 826	(AC)_8_C	7	6	86	14
UBC 809	(AG)_8_G	6	4	67	9
UBC 843	(CT)_8_RA	7	7	100	16
	Total	52	44		
	Average	6.5	5.5	83.6	12.5

R = (AG)

**Fig. 9:**
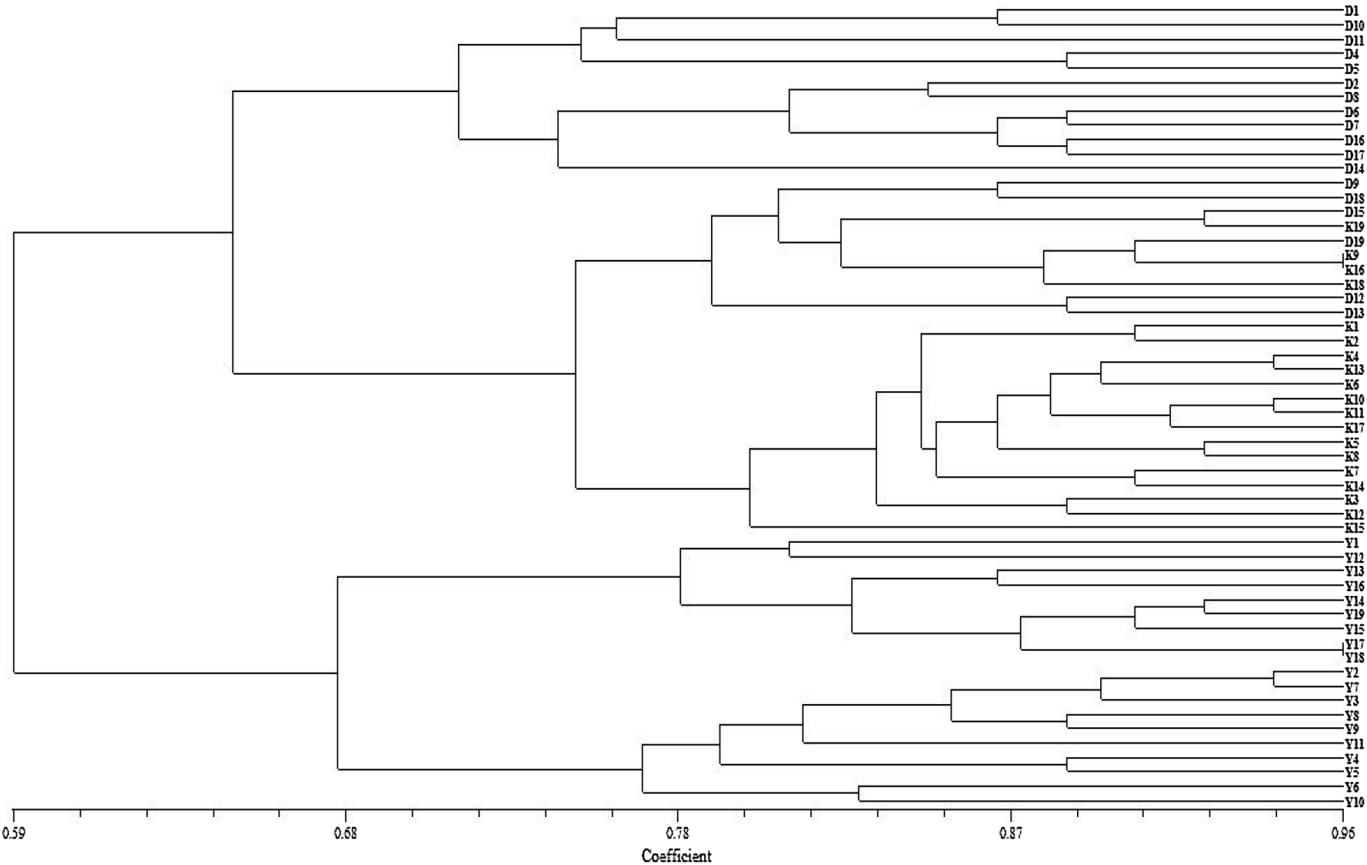
Dendrogram of all samples generated by unweighted pair group method with arithmetic averages (UPGMA) cluster analysis of genetic similarity values determined using Inter-Simple Sequence Repeats (ISSR). K1–K19, Kufranjah valley (northern population); D1–D18, Dead Sea region (northern population); Y1–Y19, Wadi al Yutm (southern population)

Mean values of genetic similarity within and between populations are shown in Table 3. The genetic similarity among individuals, calculated as Jaccard’s coefficient, was high within populations [e.g., 0.96 between K16 and K9 (Kufranjah valley, northern population) and between Y18 and Y17 (Wadi al Yutm, southern population)]. It was much lower between northern and southern populations [e.g., 0.27 between individuals Y3 (Wadi al Yutm southern population) and D15 (Dead Sea northern population)]. Individuals from the three populations clustered in two groups. The first included all samples belonging to the southern population and the second included all samples from the northern populations. While the northern group was further split into two subgroups, these did not align with population locations. One subgroup contained all samples from Kufranjah valley (K) and some from the Dead Sea valley (D), and the other contain the remaining individuals from the Dead Sea population (Fig. 9).

**Table 3:** Mean values of genetic similarity within and between *Blepharis* populations surveyed in Jordan

Population	Kufranjah (northern)	Dead Sea (northern)	al Yutm (southern)
Within	0.83	0.72	0.74
Between			
Dead Sea	0.67	–	–
Al Yutm	0.59	0.59	–

**Fig. 10:**
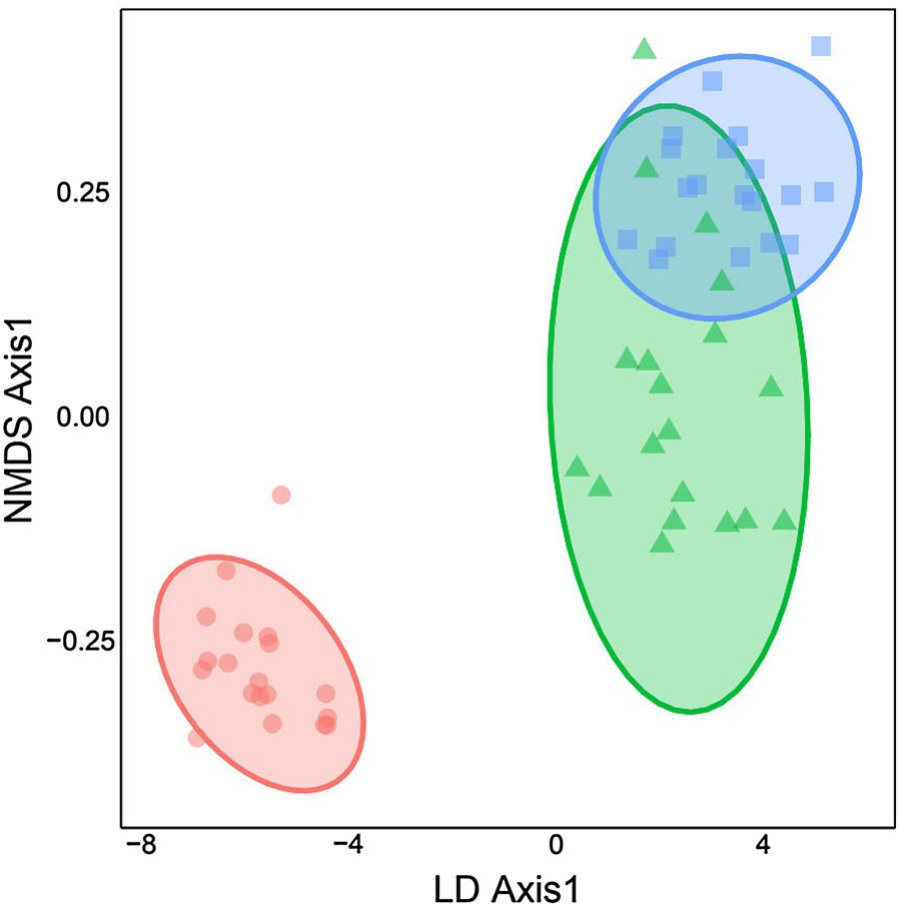
Bivariate plot showing correspondence of phenotype (x-axis) with genotype (y-axis) for individuals from Kufranjah valley (northern population, blue squares), Dead Sea region (northern population, green triangles) and Wadi al Yutm (southern population, red circles). The linear discriminant (LD) axis is based on Euclidean distances of 14 morphological characteristics standardized to a mean of zero and standard deviation of one. The nonmetric multidimensional scaling (NMDS) axis is based on Jaccard’s pairwise distances between Inter-Simple Sequence Repeats (ISSR) of genotypes

Our findings strongly support that the northern and southern populations of *Blepharis* are genetically distinct. This corresponds with the observed population-based morphological trait variation, supporting the applicability and reliability of ISSR markers for assessing genetic relatedness among closely related species with high accuracy and specificity. To visually compare morphological and genetic variation, we plotted the principal axis from the NMDS with the LD1 axis from the LDA. The resulting graph showed a clear correspondence between genetic differentiation and morphological divergence between, but not within, populations (Fig. 10). These results concurred with those of the LDA and Scheffe test analyses (see above), corroborating further population distinctiveness and genetic divergence.

### Number of *Blepharis* species in Jordan

Our study presents robust morphological and genetic evidence supporting the occurrence of two distinct *Blepharis* taxa in the Jordanian arid and semi-arid regions. We propose that the plants from the northern populations in west Irano-Turanian region and upper strip of the Sudanian zone below the sea level are *B*. *attenuata* (Fig. 1). We also propose that plants in the southern population from the lower strip of the Sudanian zone above sea level are *B*. *ciliaris*. Comparisons of the morphological traits of the three *Blepharis* populations examined in this study support Feinbrun-Dothan (1978) who recognized two species occurring within the larger region. The northern and southern populations are characterized by morphological distinctiveness and the genetic evidence from ISSR fingerprinting in this study substantiates the species designations.

Numerous morphological characters of the inflorescence were found to be taxonomically valuable at the species level. These characters included the density or laxness of the spike inflorescence, internode length, bract length and width, number of bract veins and lateral spines, length of the longest lateral spine, and the ratio of spine length to bract width. The significant contribution of these variables to discriminating *Blepharis* populations sampled from three sites corresponded with descriptions for two different *Blepharis* species (Feinbrun-Dothan 1978). Additional studies to determine the extent of both *Blepharis* species in Jordan will be worthwhile and further work on population genetics might clarify whether any subspecies exist.

### Identification key (updated from Feinbrun-Dothan 1978)

Spikes lax, spikelet bract width is shorter than length of the longest lateral spine…….. *B*. *attenuata*

Spikes dense and compact, spikelet bract width is longer than length of the longest lateral spine…….. *B*. *ciliaris*

### Taxonomic treatment

*Blepharis attenuata* Napper, Israel J. Bot. 21: 164 (1972); Feinbrun-Dothan, Fl. Palaest. 3: 219 and pl. 369 (1978); Feinbrun-Dothan and Danin, Anal. Fl. Eretz-Israel: 622 (1991), Furness, Rev. Palaeobot. and Palyn. 92: 256–265 (1996); Type: Israel, *Evenari* et al. B.1 (JUH, holotype, not seen; K, isotype!). *Acanthodium spicatum* sensu Nees in DC., Prodr. 11: 274 (1847), p.p., non Del. (1813). *Blepharis edulis* sensu Eig et al., Fl. Palestine: 340 (1931); Dinsmore in Post, Fl. Syria, Palestine and Sinai (ed. 2) 2: 317 (1933); Täckholm, Students’ Fl. Egypt: 111 (1956), p.p., non (Forssk.) Pers. (1806); *Blepharis ciliaris* sensu Napper, Israel J. Bot. 21: 165 (1972), excl. spec. ex Iran; Täckholm, Students’ Fl. Egypt (ed. 2): 502 (1974), p.p.; Feinbrun-Dothan, Fl. Palaes. 3: 218 and pl. 368 (1978); Feinbrun-Dothan and Danin, Anal. Fl. Eretz-Israel: 622 (1991), non (L.) B. L. Burtt (1956); Vollesen, *Blepharis* (Acanthaceae): A taxonomic revision 94–96 and Fig. 12 (2000).

Distribution. Egypt, the West Bank and Israel, Jordan.

*Blepharis ciliaris* (L.) B. L. Burtt, Notes Roy. Bot. Gard. Edinb. 22: 94 (1956); Vivi Täckholm, Students’ Fl. Egypt (ed. 2): 97 and pl. 175 (1974); Feinbrun-Dothan, Fl. Palaest. 3: 218 and pl. 368 (1978); Malik and Ghafoor, Fl. Pakistan. No. 188. Acanthaceae: 5 and Fig. 1 (1988), p.p. et excl. syn. Type: “Persia”, *Garcin*; Vollesen, *Blepharis* (Acanthaceae): A taxonomic revision 96–97 and Fig. 13 (2000). *Ruellia ciliaris* L., Sys. Nat. Ed. 12, 2: 424 (1767); L., Mant. 89 (1767) and Mant. Prior. Addit.: 515 (1771). *R*. *persica* Burm. f., Fl. Ind.: 135 and Tab. 42, Fig. 1 (1768). *B*. *edulis* (Forssk.) Pers., Syn. Pl. 2: 180 (1806); Boiss., Fl. 4: 520; Post, Fl. 2: 317; sensu T. Anderson, J. Linn. Soc. 9: 500 (1867), p.p.; C. B. Clarke in Hooker, Fl. Brit. Ind. 4: 479 (1884), p.p. and in Thiselton-Dyer, Fl. Trop. Afr. 5: 102 (1889), p.p.; *B*. *persica* (Burm. f.) O. Kuntze, Revis. Gen. 2: 483 (1891); Rechinger, Fl. Iran., Acanthaceae: 2 (1966), excl. syn; Stewart in Nasir and Ali, Ann. Cat. Vasc. Pl. W. Pak. and Kashmir: 674 (1972); *Acanthus edulis* Forssk., Fl. Aegypt.-Arab. 115. (1775). *A*. *pectinatus* Willd. ex Nees, Prodr. [A. P. de Candolle] 11: 274 (1847). *A*. *tetragonus* R.Br., Verm. Bot. Schr. 1:249 (1825).

Distribution. Egypt, the West Bank and Israel, Jordan, Saudi Arabia, Oman, Iran, Pakistan.

### Authors’ opinion and justification

The significant discrimination in morphological characters (particularly those of the inflorescence) among the sampled *Blepharis* populations from Jordan (as a test region of the Middle East) and the genetic evidence from ISSR supports the designations of the two species in this study. Our findings are consistent with descriptions for two different *Blepharis* species previously recognized by Feinbrun-Dothan (1978). Our results do not support Vollesen’s (2000) treatment of *B*. *ciliaris* sensu Napper, Israel J. Bot. 21: 164 (1972) as a synonym of *B*. *attenuata* and the hypothesis that the genus is represented in the area by a single species. Despite the broad ecological distribution range of *Blepharis* in Jordan, we strongly believe that the sampling sites, where the genus was abundant, and the data from this study were unambiguously sufficient and indicative of the natural occurrence of two distinct *Blepharis* species in the area.

## Abbreviations

ISSR: Inter-Simple Sequence Repeat
LDA: linear discriminant analysis
*lm*: linear models
NMDS: non-metric multidimensional scaling
PCR: polymerase chain reaction
RAPD: Random Amplified Polymorphic DNA
SAHN: sequential agglomerative hierarchical non-overlapping clustering
UPGMA: unweighted pair group method with arithmetic averages
